# Better Discrimination, Unchanged Practice: Are Machine Learning Models Ready to Replace Established Risk Scores in Cardiac Surgery? A Narrative Review

**DOI:** 10.3390/life16081371

**Published:** 2026-08-20

**Authors:** Dimitrios E. Magouliotis, Serge Sicouri, Vasiliki Androutsopoulou, Prokopis-Andreas Zotos, Massimo Baudo, Basel Ramlawi

**Affiliations:** 1 Department of Cardiac Surgery Research, Lankenau Institute for Medical Research, Wynnewood, PA 19096, USA; sicouris@mlhs.org (S.S.); massimo.baudo@icloud.com (M.B.); ramlawib@mlhs.org (B.R.); 2 Department of Cardiothoracic Surgery, Faculty of Medicine, University of Thessaly, Biopolis, 41110 Larissa, Greece; androutsopoulouvasiliki@uth.gr (V.A.); zotospro@hotmail.com (P.-A.Z.); 3 Department of Cardiac Surgery, Lankenau Medical Center, Wynnewood, PA 19096, USA

**Keywords:** machine learning, cardiac surgery, risk prediction, EuroSCORE II, STS score, calibration, external validation, clinical decision support

## Abstract

Preoperative risk stratification underpins consent, treatment selection, and quality benchmarking in cardiac surgery, a task served for two decades by regression-derived scores such as the European System for Cardiac Operative Risk Evaluation II (EuroSCORE II) and the Society of Thoracic Surgeons Predicted Risk of Mortality (STS PROM), together with procedure-specific tools. A rapidly expanding literature reports that machine learning (ML) models achieve higher discrimination than these scores, yet established scores remain the instruments actually used at the bedside. This narrative review examines that paradox. Drawing on studies emphasized between 2023 and 2026, we argue that the reported advantage of ML is real but modest. This advantage is driven primarily by improved discrimination, while key measures of clinical value, including calibration, net benefit, and external or temporal validation, are infrequently reported. We organize the evidence around a four-lens appraisal (discrimination, calibration, clinical utility, and generalizability) and show that most cardiac surgery ML studies focus only on discrimination. We then consider why superior discrimination has not changed practice and outline the evidence needed for an ML-based risk model to justify replacing an established scoring system. The current literature supports a measured conclusion: ML is a discrimination upgrade in search of clinical proof.

## 1. Introduction

Risk stratification is embedded in nearly every stage of the cardiac surgical pathway. Preoperative estimates of mortality and major morbidity inform patient consent, guide the choice between surgical, transcatheter, and medical management, calibrate expectations for intensive care and resource use, and provide the case-mix adjustment that underpins institutional benchmarking and public quality reporting [1,2]. For more than two decades this task has been served by regression-derived risk scores, chiefly the European System for Cardiac Operative Risk Evaluation II (EuroSCORE II) and the Society of Thoracic Surgeons Predicted Risk of Mortality (STS PROM), alongside more parsimonious and procedure-specific tools developed for particular clinical settings [1,2,3,4,5,6,7].

Over the past several years, machine learning (ML) has been proposed as the natural successor to these scores. A large and fast-growing body of work reports that gradient boosting, random forests, and neural networks achieve higher discrimination than the established models, most often expressed as a gain in the area under the receiver operating characteristic curve (AUC) [8,9]. The framing is intuitive. Established scores rest on additive or logistic regression with a fixed and relatively small set of predictors, whereas flexible learners can in principle capture nonlinearities and interactions that regression omits. If discrimination were the whole story, the conclusion would be straightforward, and these models would already be displacing their predecessors in preoperative clinics and multidisciplinary meetings.

A brief clarification of terminology is warranted, since the two terms are often used interchangeably. Machine learning is the general class of algorithms that learn statistical relationships directly from data rather than from coefficients specified in advance, and it includes penalized regression, tree ensembles, and kernel methods. Deep learning is a subset of machine learning that uses neural networks with multiple hidden layers to learn hierarchical representations of the input, and it typically requires substantially larger datasets to do so reliably. Throughout this review we use machine learning as the umbrella term and reserve deep learning for models built on multilayer neural networks, which in cardiac surgery remain a minority of published risk models.

They are not. Established scores remain the instruments actually used in routine practice and registry reporting, while ML models remain, with few exceptions, confined to the datasets in which they were developed [10]. The gap between reported performance and clinical adoption is the central problem of this review. It is tempting to attribute the gap to conservatism or to slow regulatory pathways, but a closer reading of the literature suggests a more specific and more actionable explanation. The apparent superiority of ML is characterized almost entirely through a single lens, discrimination, while the properties that actually determine whether a risk estimate is safe and useful at the bedside are rarely measured [11,12,13,14].

The stakes of getting this right are not academic. A preoperative risk estimate has direct clinical consequences. It is quoted to patients and families during consent, and it shapes whether a borderline candidate is offered an operation or referred for a transcatheter or medical alternative. When aggregated, it also determines how surgeons and institutions are compared and remunerated [1,2]. An estimate that is well ranked but numerically wrong can systematically deny surgery to patients who would benefit or expose others to operations they should have avoided, and it can distort benchmarking in ways that penalize the very units that take on the sickest patients. The proliferation of ML models in this space, many published with impressive headline metrics but limited evaluation beyond discrimination, raises concerns that extend beyond methodology and into patient safety. The key question is not whether ML can, in principle, predict cardiac surgical outcomes, but whether any given ML model has demonstrated sufficient reliability, validity, and safety to support clinical decision-making.

This review is deliberately framed as a critical appraisal of evidence quality rather than a catalog of models. Its guiding question is whether ML risk models are ready to replace established scores in cardiac surgery once they are judged on more than discrimination. We first describe the established scores as a heterogeneous class, because the common shorthand of a single incumbent obscures important differences that shape any fair comparison. We then summarize the incremental contribution of ML before appraising the evidence through four domains: discrimination, calibration, clinical utility, and generalizability. We also consider that algorithmic bias is a cross-cutting issue that influences performance and applicability across all four domains.

Finally, we consider why better discrimination has not translated into changed practice and outline the evidence that would justify adoption.

Throughout, we argue that the honest reading of the current literature does not yet support replacing established scores and that the burden now lies less in improving discrimination than in demonstrating calibration, validation, fairness, and clinical benefit.

The novelty of this review lies in how the evidence is organized rather than in the identification of new studies. Existing syntheses have addressed adjacent but different questions. A systematic review and meta-analysis pooled discrimination across mortality models and concluded that machine learning improves the area under the curve relative to conventional scores [8]. Broader narrative overviews have surveyed the range of artificial intelligence applications across cardiovascular care [15]. Methodological appraisals conducted outside cardiac surgery have documented pervasive risk of bias and incomplete reporting in machine learning prediction studies [16,17]. What none of these provides is an appraisal of cardiac surgical risk models against the specific criteria that would have to be satisfied before an established score could responsibly be replaced at the point of care. The contribution of this review is therefore the four-lens framework introduced in Section 5, which evaluates discrimination, calibration, clinical utility, and generalizability as separate and cumulative requirements, together with a tabulation showing which of these dimensions representative published models actually report. This reframes the question from whether machine learning predicts better to whether it has been shown to be safe to act upon.

## 2. Methods

**Figure 1:**
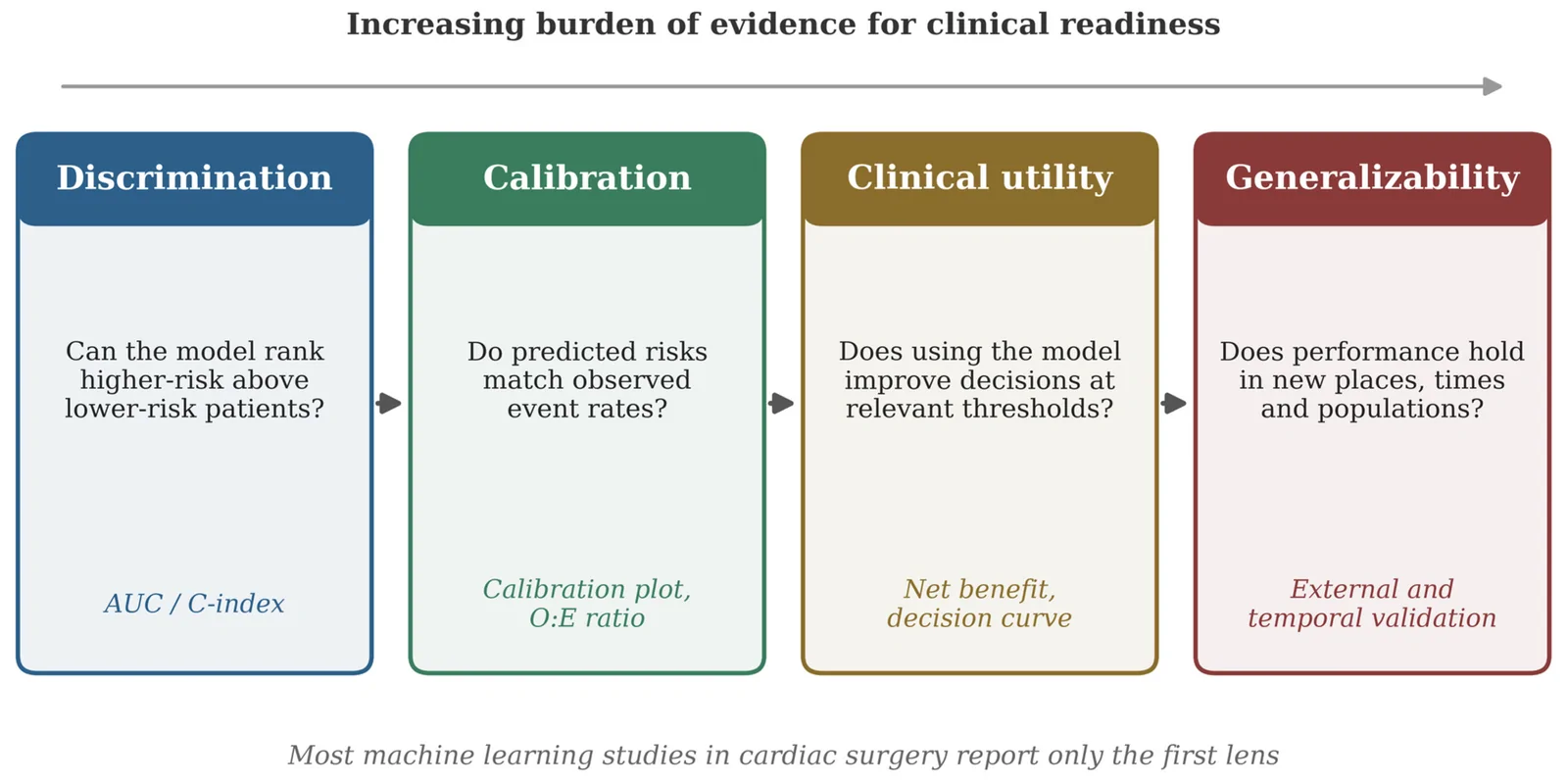
A four-lens framework for appraising machine learning risk models in cardiac surgery. Each lens answers a distinct question and imposes an increasing burden of evidence for clinical readiness. This schematic is conceptual and is intended to organize the appraisal rather than to present empirical data. This figure was created in BioRender (8 July 2026). Magouliotis, D. (2026).

**Figure 2:**
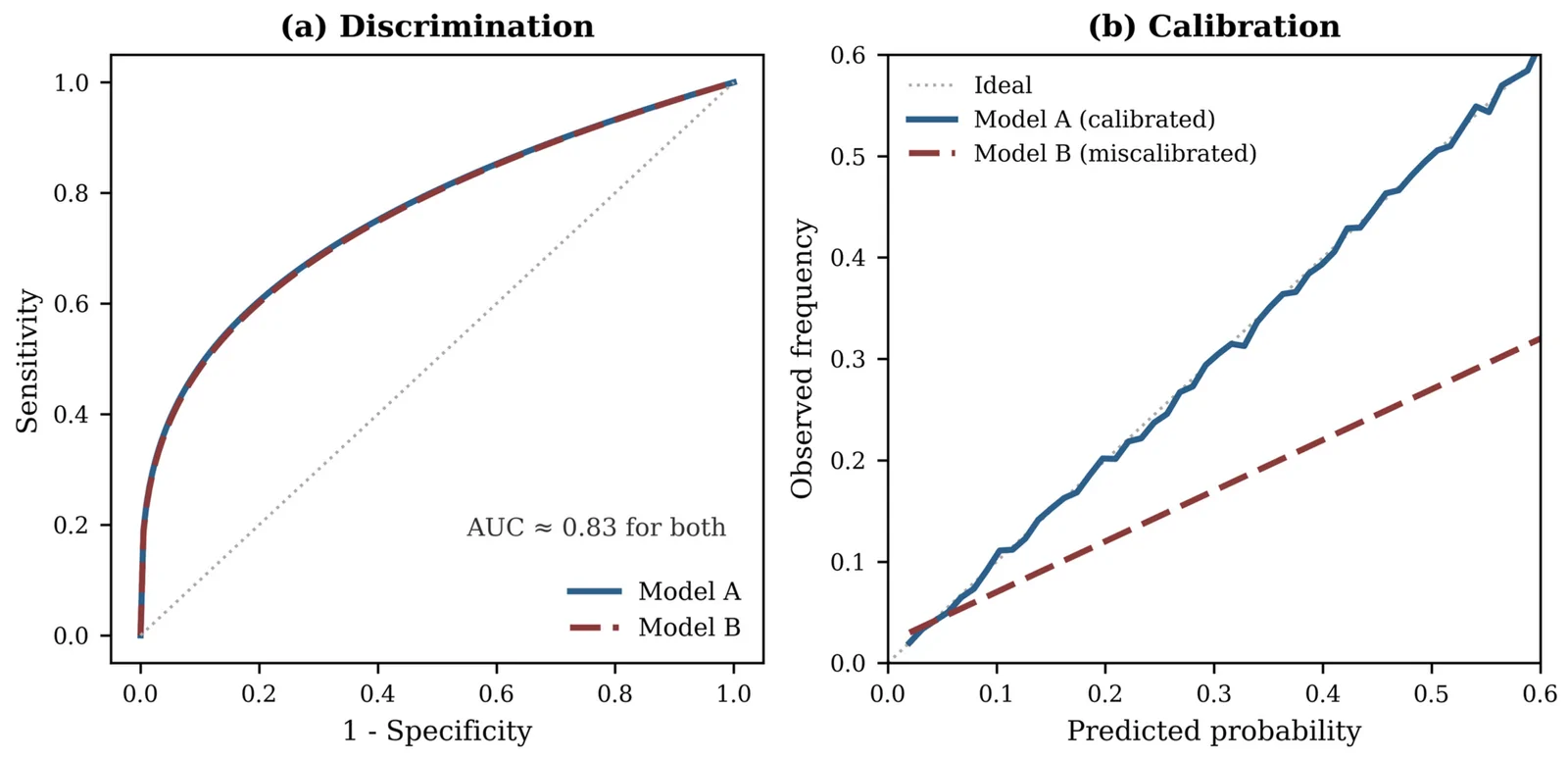
Discrimination and calibration are independent properties. (**a**) Two models with nearly identical receiver operating characteristic curves (area under the curve, AUC) and therefore equivalent discrimination. Dashed line represents the ideal Discrimination (**b**) The same two models can differ markedly in calibration, with one closely following the ideal line while the other systematically misestimates risk. The curves are illustrative and do not represent a specific dataset. This figure was created in BioRender (8 July 2026). Magouliotis, D. (2026).

This is a narrative review. We searched PubMed and Embase for studies addressing ML risk prediction in adult cardiac surgery, with emphasis on work published between 2023 and 2026, while incorporating seminal earlier studies when relevant to the argument. Search terms combined concepts of machine learning and artificial intelligence with cardiac surgery, operative risk, and the established risk scores, including EuroSCORE II, STS PROM, ACEF, GERAADA, and the Penn classification. We prioritized primary studies that compared ML models directly against established scores and studies or methodological guidance that reported calibration, net benefit, or decision-curve analysis, or external and temporal validation, since these dimensions are central to the review’s argument. Methodological reference points for prediction-model appraisal, including the TRIPOD and PROBAST families of guidance, were included to frame the analysis [12,13,16,18,19,20]. Given the narrative design, this synthesis does not claim exhaustive coverage and does not pool results quantitatively; it is intended to characterize the state and the limits of the current evidence rather than to estimate a summary effect. Where the literature is heterogeneous, we describe the direction and consistency of findings rather than a single pooled estimate. We searched PubMed/MEDLINE and Embase from database inception to May 2026, with deliberate emphasis on work published between 2023 and 2026. The search combined three concept blocks with the Boolean operator AND, each block combined internally with OR: (i) (‘machine learning’ OR ‘artificial intelligence’ OR ‘deep learning’ OR ‘neural network’ OR ‘random forest’ OR ‘gradient boosting’ OR ‘XGBoost’ OR ‘support vector machine’); (ii) (‘cardiac surgery’ OR ‘cardiac surgical procedures’ OR ‘coronary artery bypass’ OR ‘valve surgery’ OR ‘aortic surgery’); and (iii) (‘risk prediction’ OR ‘risk score’ OR ‘operative mortality’ OR ‘EuroSCORE’ OR ‘STS score’ OR ‘ACEF’ OR ‘GERAADA’ OR ‘Penn classification’ OR calibration OR ‘external validation’ OR ‘decision curve’). Reference lists of retrieved articles and relevant methodological guidance were screened manually for additional sources. Studies were eligible if they were published in English, concerned adult cardiac surgery, and either developed or validated a machine learning model for a perioperative outcome, compared such a model against an established risk score, or provided methodological guidance directly relevant to the appraisal of prediction models. We excluded studies confined to pediatric or congenital populations, studies addressing purely diagnostic imaging tasks without an outcome prediction component, conference abstracts and preprints without peer review, and studies reporting no performance metric. Records were screened by title and abstract and then in full text by two authors, with disagreements resolved by discussion. Because the aim was to characterize the state and the limits of the evidence rather than to estimate a summary effect, this review is narrative rather than systematic: it was not registered, it does not follow PRISMA, no formal risk-of-bias instrument was applied to each record, and results are not pooled. Studies selected for providing evidence in Table 1 and for tabulation in Table 2 were chosen to span the range of cohort sizes, outcomes, algorithm families, and appraisal practices in the field rather than to constitute an exhaustive sample, and this purposive selection is a limitation. Figure 1 and Figure 2 are conceptual schematics prepared by the authors to organize the argument of this review; neither is derived from patient-level data, and neither reproduces a previously published figure. Figure 1 is a diagrammatic framework. The receiver operating characteristic and calibration curves shown in Figure 2 are illustrative curves generated numerically for demonstration purposes only, in order to show that discrimination and calibration are mathematically independent properties; they do not represent any patient cohort, and no statistical analysis of clinical data was performed for this review. Both figures were produced in Python 3.11 using the Matplotlib 3.11.1 and NumPy 2.5 libraries.

## 3. Established Risk Scores in Cardiac Surgery: A Heterogeneous Class

A fair comparison between ML and established scores begins with the recognition that the established tools are not a single, uniform benchmark. They differ in derivation, in the outcome they target, in the breadth of procedures they cover, and even in whether they produce a probability at all. Collapsing them into a generic incumbent, as many ML studies implicitly do when they benchmark only against EuroSCORE II, is itself a source of misleading comparison [1,9].

The two most widely used tools are general adult-cardiac probability scores. EuroSCORE II, derived from more than 22,000 patients across 43 countries, updated the original EuroSCORE to reflect improved contemporary outcomes and remains one of the most frequently applied models worldwide [1]. The STS PROM is derived from hundreds of thousands of records in the STS Adult Cardiac Surgery Database. It is procedure-specific, providing separate models for isolated coronary artery bypass grafting, isolated valve surgery, and combined valve plus bypass procedures, together with separate endpoints for mortality and several major morbidities [2,3]. Both are logistic regression models that output a calibrated probability. Both were carefully validated at derivation, and both are subject to the same fundamental vulnerability: as surgical outcomes improve and case mix shifts, a model estimated on historical data drifts out of calibration, tending to overestimate risk in contemporary low-risk patients [1,2,3]. This is not a defect unique to regression; it is a property of any fixed model applied to a moving population, and it is precisely why the STS models are periodically re-estimated and why EuroSCORE II has attracted a substantial recalibration literature [21].

A second family is defined by parsimony. The age, creatinine, and ejection fraction (ACEF) score reduces preoperative risk assessment to three variables and was originally developed for elective cardiac surgery and later extended to percutaneous and transcatheter settings [4]. ACEF II added hematocrit and an emergency term to improve performance in higher-risk and non-elective cases while preserving the law-of-parsimony philosophy [5]. These scores trade discrimination for simplicity and transparency, and they perform best in the low-to-intermediate-risk elective populations for which they were designed [4,5].

The distinction between a computed probability and an ordinal classification is merely semantic; it fundamentally affects how ML models should be compared with traditional risk scores.

A probability score can be assessed for calibration and can be fed directly into a decision-analytic calculation; an ordinal classification cannot, but it encodes clinically salient structure, such as the presence of malperfusion, that a probability may obscure. When an ML model is said to outperform a classification, the claim is often about discrimination in a specific cohort, and it does not follow that the model would better support the decision the classification was designed to inform. Comparisons between these fundamentally different types of tools require careful interpretation, a distinction that is not always adequately addressed in the literature.

The transcatheter era has further complicated the picture. As aortic valve disease is increasingly managed percutaneously, the established surgical scores are applied to populations and decisions for which they were never derived, and their calibration in these settings is uncertain [2,22]. This is fertile ground for ML, but it is also a setting where the temptation to benchmark against a score used outside its intended domain is strongest, and where an apparent ML advantage may again reflect a weak comparator rather than a strong model.

A third family is procedure and population-specific, and it is here that the heterogeneity of the incumbents becomes most consequential. In acute type A aortic dissection, general scores such as EuroSCORE II substantially underestimate operative mortality, which is why dissection-specific tools were developed [6,22]. The German Registry of Acute Aortic Dissection Type A (GERAADA) score predicts 30-day mortality from nine preoperative variables and has been externally validated across Europe, Asia, and the United States [6].

The Penn classification differs fundamentally from conventional risk scores. It is an ordinal clinical classification that stratifies patients by the presence and extent of ischemia and malperfusion, predicting mortality without generating an individualized probability estimate [7]. The contrast matters for any ML comparison. A learner benchmarked against EuroSCORE II in a dissection cohort is competing against a model already known to be poorly calibrated for that population, whereas a comparison against GERAADA or the Penn classification is a far more demanding test [6,7,22]. Table 1 summarizes this heterogeneity.

## 4. What Machine Learning Added: The Discrimination Story

The case for ML in cardiac surgery rests primarily on discrimination. Across a range of outcomes and cohorts, flexible learners have reported modest but reasonably consistent improvements in AUC relative to established scores.

The most instructive evidence comes from large, well-powered comparisons in which the same variables are made available to every model. In an analysis of more than 220,000 adults undergoing cardiac surgery in a national database, gradient boosting and random forest models achieved an AUC of approximately 0.83 for in-hospital mortality, compared with approximately 0.82 for EuroSCORE II, a small but real gain in ranking performance [9]. A systematic review and meta-analysis of mortality prediction likewise concluded that ML tends to improve discrimination over conventional scores, although the pooled advantage was incremental rather than transformative [8].

Two features of this evidence deserve emphasis, because they are frequently lost when a single headline AUC is quoted. First, the magnitude of the gain is small. Differences in AUC of one to two hundredths, while sometimes statistically significant in very large samples, correspond to modest changes in the practical task of separating higher-risk from lower-risk patients, and their clinical importance cannot be inferred from the AUC alone [9,14]. Second, and more fundamentally, the advantage of complex learners over well-specified regression is not guaranteed. A broad systematic review of clinical prediction models found no consistent performance benefit of ML over logistic regression when both were developed and evaluated to comparable standards [10]. This finding reframes the discrimination gains reported in cardiac surgery. They appear to depend on data volume and modeling care rather than on the algorithm itself.

There is a principled reason to expect the gains to be modest in this particular setting. The established scores operate on structured, tabular, expert-curated preoperative variables, and on such data the relationships between predictors and operative mortality are largely captured by additive and low-order terms that regression already models well. The additional signal available to a flexible learner, in the form of high-order interactions and nonlinearities, is real but limited, which is consistent with the broad finding that ML offers no reliable advantage over logistic regression for tabular clinical prediction [10]. Where ML has a clearer theoretical edge is with data that regression handles poorly, such as waveform, imaging, and other high-dimensional or unstructured inputs, and with very large, linked datasets that can support and revalidate complex models over time [15]. The lesson is not that ML is unhelpful, but that the specific task of improving a tabular preoperative score is close to the setting in which its advantage is smallest.

The national-database comparison is particularly instructive in this regard. When the same investigators examined not only discrimination but also calibration and clinical utility, the picture became more nuanced. The ML models did not improve calibration relative to EuroSCORE II, and although decision-curve analysis suggested a net-benefit advantage for the boosted models, the authors concluded that the clinical impact of the improvement was modest [9]. This is a representative pattern rather than an isolated result. The discrimination story, read carefully, is a story of small and conditional gains, It is only the first of several dimensions that determine whether a model can be trusted.

Because ‘machine learning’ is often used as a single label for a heterogeneous set of methods, it is worth stating precisely which model families dominate this literature, since their inductive biases differ and their failure modes are not identical. Four families account for most published cardiac surgery risk models. Penalized regression, including LASSO and elastic net, retains the additive structure of logistic regression while shrinking coefficients to limit overfitting, and it is therefore the family closest to the established scores. Tree ensembles, comprising random forests and gradient boosted trees such as XGBoost, aggregate many decision trees and capture nonlinearities and interactions automatically; they are the most frequently reported family and usually the best performing on tabular preoperative data. Kernel methods, principally the support vector machine, separate classes in a transformed feature space. Neural networks, ranging from shallow multilayer perceptrons to deep architectures adapted for tabular input, learn hierarchical representations and require the largest datasets to do so reliably [23,24,25].

Predictor sets also vary in a way that materially affects comparability. Some models are restricted to the preoperative variables available to the established scores, which permits a fair head-to-head comparison, whereas others incorporate intraoperative or early postoperative data such as hemodynamic time series, transfusion requirements, or ventilation duration [23,26,27]. Models of the latter type may report higher discrimination, but they answer a different clinical question and cannot substitute for a preoperative score used to decide whether to operate.

Table 2 summarizes representative published machine learning models in cardiac surgery, specifying for each the cohort and sample size, the outcome, the algorithms compared, the predictor set, the discrimination achieved relative to the comparator, and whether calibration and external validation were reported [9,23,24,25,26,27].

**Table 2:** Representative published machine learning models for outcome prediction in cardiac surgery, showing sample size, predicted outcome, algorithms compared, predictor set, discrimination relative to the comparator, and whether calibration and external validation were reported. AKI, acute kidney injury; ANN, artificial neural network; AUC, area under the receiver operating characteristic curve; CABG, coronary artery bypass grafting; CI, confidence interval; DCA, decision curve analysis; GBM, gradient boosting machine; LR, logistic regression; ML, machine learning; RF, random forest; STS, Society of Thoracic Surgeons; SVM, support vector machine; XGBoost, extreme gradient boosting.

Study	Sample Size and Setting	Outcome	Algorithms Compared	Predictor Set	Discrimination (AUC) vs. Comparator	Calibration Reported	External Validation
Sinha et al., 2023 [9]	227,087; UK national registry	In-hospital mortality	XGBoost, RF, LR vs. EuroSCORE II	Preoperative registry variables shared with EuroSCORE II	~0.83 vs. ~0.82	Yes; DCA also reported	No
Allou et al., 2023 [25]	165,640; 43 French centers	In-hospital mortality	Tabular transformer, RF, XGBoost vs. EuroSCORE II	Preoperative variables from a national prospective database	0.834 (95% CI 0.831–0.838) vs. lower for EuroSCORE II	Not reported; DCA reported	No
Ong et al., 2023 [24]	7745; procedures without an STS score, 3 US centers	Operative mortality	Elastic net LR, SVM, RF, XGBoost	Preoperative variables from the institutional STS dataset	0.83 (RF)	Yes; calibration slope and expected-to-observed ratio 1.00	Yes; 2 external hospitals (0.81 and 0.79)
Fan et al., 2022 [23]	5443; single center	Postoperative mortality	RF, ANN, SVM, GBM vs. EuroSCORE I and II, STS, LR	Preoperative and intraoperative variables	0.87 (RF) vs. 0.70–0.74	Not reported	No
Behnoush et al., 2023 [27]	8493; hypertensive patients undergoing CABG	1-year mortality	LR, RF, ANN, XGBoost, naive Bayes	11 selected baseline and periprocedural features, led by ventilation hours and ejection fraction	0.82, highest for LR rather than any ML model	Not reported	No
Tseng et al., 2020 [26]	671; single center	Cardiac surgery associated AKI	LR, SVM, RF, XGBoost, RF plus XGBoost ensemble	Demographics, preoperative biochemistry and medication, intraoperative hemodynamic time series	0.843 (ensemble); 0.839 (RF)	Not reported	No

Read quantitatively, these studies show a consistent pattern. Sample sizes span more than two orders of magnitude, from 671 to 227,087 patients. The best-performing machine learning model in each study achieved an area under the curve between 0.82 and 0.87, against comparator scores ranging from 0.70 to 0.82. The incremental gain is therefore real but generally confined to a few hundredths. It is largest in the smaller single-center cohorts, where optimism is hardest to exclude. In the largest multicenter analyses the margin over EuroSCORE II narrows to approximately 0.01 to 0.02. Against this, calibration was explicitly reported in only two of the six studies, decision-curve analysis in two, and external validation in one. Notably, in one study the best discrimination was achieved by logistic regression rather than by any machine learning model [27]. The asymmetry between universally reported discrimination and sporadically reported calibration, utility, and generalizability is the central quantitative observation of this review.

### 4.1. Beyond Tree Ensembles: Transformers, Foundation Models, and Multimodal Learning

The literature summarized above is dominated by tree ensembles and shallow neural networks, but the methodological frontier has moved. Transformer architectures, originally developed for sequence modeling, have been adapted to tabular clinical data, and the largest cardiac surgical comparison to date used a tabular transformer that outperformed both random forests and gradient boosting on discrimination and net benefit [25]. Beyond this, three directions are likely to shape the next generation of models. Foundation models, trained by self-supervision on large and heterogeneous corpora and then adapted to specific tasks with little labeled data, promise to reduce the dependence on curated registry variables that constrains current risk scores [28]. Multimodal learning seeks to combine structured registry data with imaging, waveform, genomic, and free-text sources, which is precisely the setting in which flexible learners hold a genuine theoretical advantage over tabular regression [29]. Graph-based approaches, in which patients, vessels, or care pathways are represented as nodes and edges rather than as independent rows, offer a natural encoding of anatomical and relational structure that conventional models discard.

Two cautions are warranted. First, at the time of writing these approaches remain largely unrepresented in published adult cardiac surgical risk prediction, where the evidence base is still overwhelmingly tabular; the promise is therefore extrapolated from adjacent fields rather than demonstrated in this one. Second, and more important for the argument of this review, greater architectural sophistication does not by itself address any of the four appraisal dimensions. A foundation model that is not calibrated, not externally validated, and not shown to improve decisions is no more ready for the bedside than a random forest with the same deficiencies. If anything, larger and more opaque models raise the evidentiary bar, because their failure modes are harder to anticipate and their behavior under distribution shift is less predictable [30].

### 4.2. Explainable Artificial Intelligence and Clinical Adoption

The opacity of flexible learners has motivated a parallel literature on explainable artificial intelligence, which is directly relevant to clinical adoption because a surgeon cannot reasonably act on a risk estimate that cannot be interrogated. Two model-agnostic methods dominate practice. SHapley Additive exPlanations assign each input variable a contribution to an individual prediction using a game-theoretic allocation, yielding both local explanations for single patients and global importance rankings [31]. Local interpretable model-agnostic explanations instead fit a simple surrogate model in the neighborhood of a single prediction, approximating locally what the complex model does [32]. In cardiac surgery, SHAP is now routinely reported, and has been used to identify the dominant contributors to predicted mortality and to acute kidney injury, including intraoperative urine output, transfusion requirement, ventilation duration, and ejection fraction [23,26,27].

These methods make a genuine contribution to clinical acceptability, since they support case-level discussion in the multidisciplinary meeting and allow implausible model behavior to be detected. Their limitations must nevertheless be stated plainly. Post hoc explanations are approximations of the model rather than descriptions of causal mechanism, and they can be unstable, with different methods or different perturbation schemes yielding different attributions for the same patient. An explanation of why a model produced a number does not establish that the number is correct. Explainability therefore belongs alongside the four lenses rather than within them: it is a prerequisite for trust and for meaningful clinical scrutiny, but it is not a substitute for calibration, net benefit, or external validation [14,30].

## 5. From Discrimination to Clinical Value: A Four-Lens Appraisal

Discrimination answers a narrow question: can the model place higher-risk patients above lower-risk patients in rank order? It says nothing about whether the predicted probabilities are numerically correct, whether acting on them improves decisions, or whether performance holds outside the development sample. These are distinct properties, and each corresponds to a lens through which a risk model must be viewed before it can be called ready for practice (Figure 1). The central weakness of the current cardiac surgery ML literature is not that it fails these tests but that, for the most part, it rarely applies them.

### 5.1. Discrimination

Discrimination is necessary but far from sufficient. A model with excellent ranking performance can still be dangerous if its probabilities are systematically wrong, and two models with essentially identical AUC can behave very differently when their outputs are used to make decisions [14]. The reliance on AUC as the primary and often sole endpoint reflects convenience as much as principle: it is a single number, it is threshold-free, and it is easy to compare. But it is also insensitive to errors that matter most in surgical decision-making, where the absolute predicted risk, not merely its rank, informs whether a patient is offered an operation. Literature that reports discrimination alone has, in effect, measured the least decision-relevant property of its models.

### 5.2. Calibration

Calibration describes the agreement between predicted probabilities and observed event rates. A well-calibrated model that predicts a 10 percent risk is correct if approximately 10 percent of such patients die; a miscalibrated model may discriminate perfectly yet assign probabilities that are uniformly too high or too low, or too extreme at the tails. Calibration has been called the Achilles heel of predictive analytics precisely because it is frequently ignored at development and validation, and because poor calibration is not a cosmetic flaw but a source of misleading and potentially harmful risk estimates [14]. Figure 2 illustrates that discrimination and calibration are independent: two models with nearly identical ROC curves that can diverge sharply in calibration, and the miscalibrated model will misinform any decision that depends on the absolute risk.

The relevance to cardiac surgery is direct. The established scores earned their place partly through attention to calibration and its maintenance over time: the STS models are periodically re-estimated, and EuroSCORE II generated extensive recalibration literature precisely because calibration drift was recognized and addressed [2,3,21]. By contrast, systematic appraisals of ML prediction models across medicine have repeatedly found that calibration is the most commonly omitted performance measure, frequently absent even from studies claiming external validation [16]. A model that has never been shown to be calibrated cannot be trusted to provide an absolute estimate of operative risk, regardless of how high its AUC may be.

### 5.3. Clinical Utility and Net Benefit

Even a discriminating and well-calibrated model has clinical value only if it improves decisions relative to the alternatives, which include the current standard score and simple default strategies such as treating all or no patients. Decision-curve analysis was developed to answer this question by quantifying net benefit across the range of decision thresholds clinically plausible [33]. Net benefit integrates the consequences of true and false positives at a given threshold and therefore speaks directly to the decision the clinician actually faces, unlike discrimination or calibration in isolation. Miscalibration, notably, reduces net benefit, which is one reason the three lenses cannot be separated: a model that is poorly calibrated will generally deliver less clinical benefit than its discrimination would suggest [14]. In the cardiac surgery ML literature, decision-curve analysis remains the exception rather than the rule, and where it has been performed, as in the large national-database comparison, the net-benefit advantage of ML over the established score was judged modest [9]. The practical implication is stark: for most published ML models in this field, there is no evidence that using them would improve clinical decision-making.

### 5.4. Generalizability and External Validation

The final lens is generalizability. A model’s performance in the data on which it was developed, even under cross-validation, systematically overstates how it will perform elsewhere; this is because it partly reflects idiosyncrasies of the development sample. Meaningful evidence requires external validation in geographically or temporally distinct cohorts, and ideally, temporal validation that tests robustness to the very calibration drift that affects the established scores [11,13]. This is where the ML literature is the weakest. Broad systematic reviews of ML prediction-model studies have found that external validation is uncommon, that reporting is frequently incomplete, and that the overall risk of bias is high, with small sample sizes and inadequate handling of missing data as recurrent contributors [16,17,30]. The experience of prediction modeling during the COVID-19 pandemic, where the overwhelming majority of proposed models were rated at high risk of bias and few were fit for deployment, is a cautionary precedent that applies with equal force to cardiac surgery [17]. Table 3 draws the four lenses together and summarizes the principal gap in the current evidence.

### 5.5. Algorithmic Bias and Equity

A fifth consideration cuts across the four lenses and is especially relevant to models used to determine surgical eligibility.

A model can be well discriminating and, on average, well calibrated, yet perform unevenly across patient subgroups defined by sex, age, race, or socioeconomic status, so that its risk estimates are systematically too high or too low for some groups [14,30]. In a surgical context this is not an abstract fairness concern: subgroup miscalibration can translate directly into inequitable access to surgery, because a model that overestimates risk in a particular group will steer its members away from operations from which they would benefit.

Flexible learners may be even more vulnerable to this failure mode than parsimonious regression models because they can absorb and amplify patterns in the training data that reflect historical inequities in access and outcomes rather than underlying biology [30]. The appropriate response is to assess calibration within clinically relevant subgroups, not merely in aggregate, and to report where a model’s performance is uneven. This is rarely done in the cardiac surgery ML literature, and its omission is one more reason that current evidence falls short of the standard required for a tool that will influence surgical selection.

## 6. The Translation Gap

If ML models discriminate at least as well as the established scores, why have they not entered practice? The four-lens appraisal supplies much of the answer. A tool cannot responsibly replace EuroSCORE II or the STS PROM at the point of care unless it can quote an absolute risk that is calibrated, has been shown to improve decisions, and holds up outside its development sample. Most published cardiac surgery ML models satisfy none of these conditions, and a model that clears only the discrimination bar is not, in any clinically meaningful sense, ready for clinical use [11,14,16].

Several specific factors compound the problem. The first is calibration neglect, already discussed, which alone disqualifies a model from quoting individual risk. The second is the scarcity of external validation, which leaves reported performance untethered from real-world generalizability [16,17]. The third is methodological fragility at development: small effective sample sizes relative to the number of candidate predictors, informal or absent sample-size justification, and inadequate handling of missing data are pervasive, and each inflates optimism and risk of bias [16,30,34]. The fourth is opacity. Many high-performing learners are difficult to interrogate, and a surgeon cannot easily explain to a patient why an opaque model has assigned a particular operative risk, a barrier that established scores, with their transparent variable lists, do not face [30]. The fifth is the near-total absence of prospective and implementation studies. Almost all evidence is retrospective and in silico; there are very few studies that deploy an ML risk model in a real preoperative workflow and measure whether decisions, and ultimately outcomes, improve.

A further and underappreciated factor is the comparison itself. Because ML models are frequently benchmarked only against EuroSCORE II, and often in populations where EuroSCORE II is known to be poorly calibrated, the reported advantage can reflect a weak comparator as much as a strong model [1,9]. The acute type A dissection setting is the clearest example: outperforming a general score that substantially underestimates dissection mortality is a low bar, and the more appropriate comparators, GERAADA or the Penn classification, are rarely used [6,7,22]. A model that has not been tested against the best available procedure-specific score has not earned the claim of superiority in that procedure.

Adoption is also shaped by factors that sit downstream of model quality. Integration into the preoperative workflow is non-trivial, since a model that requires manual entry of many variables, or that cannot draw them automatically from the electronic record, will not be used regardless of its accuracy. There is also the double-edged phenomenon of automation bias, whereby clinicians may over-trust a numerical output from an opaque system, which raises the stakes of deploying a model whose calibration and subgroup behavior are unverified [30]. Regulatory and governance expectations for clinical prediction tools have meanwhile tightened, and a model presented without external validation, calibration, or evidence of clinical benefit is unlikely to satisfy them. These considerations do not replace the four-lens appraisal; they compound it, because even a methodologically sound model must still be implementable and trustworthy in situ.

None of this implies that ML has no role. Flexible learners are well suited to high-dimensional and unstructured data, to signals that regression handles poorly, and to settings where large, linked datasets are available and can be revalidated over time [15]. The point is narrower and more disciplined: for the specific task of replacing an established preoperative risk score, the current evidence does not support adoption. The reasons are methodological rather than mysterious. and therefore, in principle, remediable.

## 7. What Readiness Would Require

It is more constructive to specify what adequate evidence would look like than to catalog deficiencies. The criteria below follow directly from the gaps identified above and are drawn from established guidance for prediction-model development and appraisal rather than proposed anew [11,12,13,18,19,20].

First, ML risk models should be reported against calibration as well as discrimination, with calibration plots and summary measures presented at every validation, since a model that cannot demonstrate calibration cannot quote an absolute operative risk [14,18]. Second, clinical utility should be assessed with decision-curve analysis against the relevant comparator so that the question is not whether the model discriminates but whether it leads to better decisions over the current standard score [33].

Third, evaluation should include external validation in geographically and temporally distinct cohorts, with temporal validation used to test robustness to the calibration drift that affects all fixed models [11,13]. Fourth, development should respect basic methodological standards, including adequate effective sample size relative to model complexity, principled handling of missing data, and transparent reporting in line with the TRIPOD and PROBAST frameworks and their AI extensions [16,18,19,20,34]. Fifth, comparisons should be made against the best available procedure-specific score and not against a general model known to be miscalibrated in the population of interest [6,7]. Finally, and most demanding of all, claims of clinical readiness should ultimately rest on prospective evaluation in a real clinical workflow, where the model informs decisions and its effect on patient care can be directly observed.

Two further principles would accelerate credible progress. The first is standardized reporting. Much of the difficulty in appraising the current literature stems from incomplete and inconsistent reporting rather than from the models themselves. The TRIPOD and PROBAST frameworks and their AI extensions were designed to remedy exactly this. Wider adherence would allow readers to judge study quality and would reduce the research waste that accompanies unusable studies [18,19,20]. The second is a shift from one-off validation to continuing evaluation. Because calibration drifts as case mix and clinical practice evolve, a model that is accurate today will not remain so indefinitely. The same maintenance discipline that keeps established scores usable, through periodic recalibration and re-estimation, must therefore be built into any ML tool intended for durable clinical use [2,3,11]. A model is not a static artifact to be validated once and deployed indefinitely but a component of care that requires ongoing surveillance.

These are not counsels of perfection. They are the ordinary requirements that established scores themselves largely met, and that any successor should be expected to meet before replacing them. A model that meets these standards would represent a genuine advance; a model that satisfies only the first would not, whatever its AUC.

## 8. Conclusions

Machine learning has demonstrated a real but modest improvement in discrimination over established risk scores in cardiac surgery. In well-powered comparisons that advantage is small and sometimes contingent on data volume and modeling care rather than on the algorithm itself. More importantly, the advantage has been characterized almost entirely through discrimination, while the properties that determine clinical value, calibration, net benefit, and external validation are seldom reported. Viewed through those additional lenses, the current evidence does not support replacing EuroSCORE II, the STS PROM, or the relevant procedure-specific scores with an ML model at the point of care. The gap between reported performance and clinical adoption reflects not a failure of nerve but a reasonable response to an incomplete evidence base.

The path forward is clear and, in principle, straightforward: report calibration, demonstrate net benefit, validate externally and over time, compare against the strongest available benchmark, and, ultimately, test prospectively. Until then, machine learning in cardiac surgical risk prediction is best understood as a discrimination upgrade in search of clinical proof.

## Figures and Tables

**Table 1:** Established preoperative risk stratification tools in cardiac surgery, illustrating the heterogeneity of the incumbent class against which machine learning models are benchmarked.

Tool	Type	Target Population or Procedure	Core Inputs	Principal Reported Limitation
EuroSCORE II	Logistic probability score	Broad adult cardiac surgery	18 variables, including age, sex, renal function, left ventricular function, pulmonary hypertension, urgency, and procedure weight	Calibration drift over time and geographic miscalibration; underestimates dissection mortality [1,21,22]
STS PROM	Logistic probability score (procedure-specific)	CABG, valve, and valve plus CABG (North America)	Extensive procedure-specific registry variable sets, including age, sex, ejection fraction, creatinine, urgency, and prior cardiac surgery	Requires periodic recalibration; limited to covered procedures [2,3]
ACEF/ACEF II	Parsimonious probability score	Elective adult cardiac surgery	3 variables: age, creatinine, and ejection fraction (ACEF II adds 2 further variables, hematocrit and emergency status)	Reduced accuracy in high-risk and non-elective settings [4,5]
GERAADA	Registry-derived probability score	Acute type A aortic dissection surgery	9 variables, including age, preoperative intubation, resuscitation, malperfusion, and hemodynamic instability	Overestimation at high-volume centers; era bias [6]
Penn classification	Ordinal clinical classification	Acute type A aortic dissection (ischemia-based)	3 ischemic presentation categories, defined by the presence and type of malperfusion (localized, generalized, or both)	Stratifies rather than quantifies risk; not a computed probability [7]

**Table 3:** A four-lens appraisal of machine learning risk models in cardiac surgery, with the question each lens answers, a representative metric, its clinical relevance, and the characteristic gap in the current literature.

Lens	Question Answered	Representative Metric	Why It Matters in Cardiac Surgery	Characteristic Gap
Discrimination	Are higher-risk patients ranked above lower-risk patients?	AUC or C-index	Screening and triage rely on separating risk groups	Reported almost universally, often as the sole endpoint [8,9]
Calibration	Do predicted risks match observed event rates?	Calibration plot, observed-to-expected ratio	Absolute risk informs consent and the decision to operate	Frequently omitted, even in external validation [14,16]
Clinical utility	Does using the model improve decisions at relevant thresholds?	Net benefit, decision-curve analysis	Determines whether the model changes management for the better	Rarely performed; benefit modest where assessed [9,33]
Generalizability	Does performance hold in new places, times, and populations?	External and temporal validation	Contemporary case mix and drift threaten any fixed model	External validation uncommon; high risk of bias [16,17,30]

## Data Availability

No new data were created or analyzed in this study. Data sharing is not applicable to this article.

## References

[1] Nashef S.A., Roques F., Sharples L.D., Nilsson J., Smith C., Goldstone A.R., Lockowandt U. (2012). EuroSCORE II. Eur. J. Cardiothorac. Surg..

[2] Shahian D.M., Jacobs J.P., Badhwar V., Kurlansky P.A., Furnary A.P., Cleveland J.C., Lobdell K.W., Vassileva C., von Ballmoos M.C.W., Thourani V.H. (2018). The Society of Thoracic Surgeons 2018 Adult Cardiac Surgery Risk Models: Part 1—Background, Design Considerations, and Model Development. Ann. Thorac. Surg..

[3] O’Brien S.M., Feng L., He X., Xian Y., Jacobs J.P., Badhwar V., Kurlansky P.A., Furnary A.P., Cleveland J.C., Lobdell K.W. (2018). The Society of Thoracic Surgeons 2018 Adult Cardiac Surgery Risk Models: Part 2—Statistical Methods and Results. Ann. Thorac. Surg..

[4] Ranucci M., Castelvecchio S., Menicanti L., Frigiola A., Pelissero G. (2009). Risk of assessing mortality risk in elective cardiac operations: Age, creatinine, ejection fraction, and the law of parsimony. Circulation.

[5] Ranucci M., Pistuddi V., Scolletta S., de Vincentiis C., Menicanti L. (2018). The ACEF II Risk Score for cardiac surgery: Updated but still parsimonious. Eur. Heart J..

[6] Czerny M., Siepe M., Beyersdorf F., Feisst M., Gabel M., Pilz M., Pöling J., Dohle D.-S., Sarvanakis K., Luehr M. (2020). Prediction of mortality rate in acute type A dissection: The German Registry for Acute Type A Aortic Dissection score. Eur. J. Cardiothorac. Surg..

[7] Patrick W.L., Yarlagadda S., Bavaria J.E., Kelly J.J., Kalva S., Grimm J.C., Rosen J.L., Ahmed S., Augoustides J.G., Szeto W.Y. (2023). The Penn Classification System for Malperfusion in Acute Type A Dissection: A 25-Year Experience. Ann. Thorac. Surg..

[8] Benedetto U., Dimagli A., Sinha S., Cocomello L., Gibbison B., Caputo M., Gaunt T., Lyon M., Holmes C., Angelini G.D. (2022). Machine learning improves mortality risk prediction after cardiac surgery: Systematic review and meta-analysis. J. Thorac. Cardiovasc. Surg..

[9] Sinha S., Dong T., Dimagli A., Vohra H.A., Holmes C., Benedetto U., Angelini G.D. (2023). Comparison of machine learning techniques in prediction of mortality following cardiac surgery: Analysis of over 220 000 patients from a large national database. Eur. J. Cardiothorac. Surg..

[10] Christodoulou E., Ma J., Collins G.S., Steyerberg E.W., Verbakel J.Y., Van Calster B. (2019). A systematic review shows no performance benefit of machine learning over logistic regression for clinical prediction models. J. Clin. Epidemiol..

[11] Steyerberg E.W., Vergouwe Y. (2014). Towards better clinical prediction models: Seven steps for development and an ABCD for validation. Eur. Heart J..

[12] Collins G.S., Reitsma J.B., Altman D.G., Moons K.G. (2015). Transparent reporting of a multivariable prediction model for individual prognosis or diagnosis (TRIPOD): The TRIPOD statement. BMJ.

[13] Moons K.G., Altman D.G., Reitsma J.B., Ioannidis J.P.A., Macaskill P., Steyerberg E.W., Vickers A.J., Ransohoff D.F., Collins G.S. (2015). Transparent Reporting of a multivariable prediction model for Individual Prognosis or Diagnosis (TRIPOD): Explanation and elaboration. Ann. Intern. Med..

[14] Van Calster B., McLernon D.J., van Smeden M., Wynants L., Steyerberg E.W., Topic Group ‘Evaluating diagnostic tests and prediction models’ of the STRATOS initiative (2019). Calibration: The Achilles heel of predictive analytics. BMC Med..

[15] Kilic A. (2020). Artificial Intelligence and Machine Learning in Cardiovascular Health Care. Ann. Thorac. Surg..

[16] Andaur Navarro C.L., Damen J.A.A.G., Takada T., Nijman S.W.J., Dhiman P., Ma J., Collins G.S., Bajpai R., Riley R.D., Moons K.G.M. (2021). Risk of bias in studies on prediction models developed using supervised machine learning techniques: Systematic review. BMJ.

[17] Wynants L., Van Calster B., Collins G.S., Riley R.D., Heinze G., Schuit E., Bonten M.M.J., Dahly D.L., Damen J.A., Debray T.P.A. (2020). Prediction models for diagnosis and prognosis of covid-19: Systematic review and critical appraisal. BMJ.

[18] Wolff R.F., Moons K.G.M., Riley R.D., Whiting P.F., Westwood M., Collins G.S., Reitsma J.B., Kleijnen J., Mallett S., PROBAST Group (2019). PROBAST: A Tool to Assess the Risk of Bias and Applicability of Prediction Model Studies. Ann. Intern. Med..

[19] Collins G.S., Dhiman P., Andaur Navarro C.L., Ma J., Hooft L., Reitsma J.B., Logullo P., Beam A.L., Peng L., Van Calster B. (2021). Protocol for development of a reporting guideline (TRIPOD-AI) and risk of bias tool (PROBAST-AI) for diagnostic and prognostic prediction model studies based on artificial intelligence. BMJ Open.

[20] Collins G.S., Moons K.G.M., Dhiman P., Riley R.D., Beam A.L., Van Calster B., Ghassemi M., Liu X., Reitsma J.B., van Smeden M. (2024). TRIPOD+AI statement: Updated guidance for reporting clinical prediction models that use regression or machine learning methods. BMJ.

[21] Collins G.S., Altman D.G. (2013). Design flaws in EuroSCORE II. Eur. J. Cardiothorac. Surg..

[22] Czerny M., Grabenwöger M., Berger T., Aboyans V., Della Corte A., Chen E.P., Desai N.D., Dumfarth J., Elefteriades J.A., Etz C.D. (2024). EACTS/STS Guidelines for Diagnosing and Treating Acute and Chronic Syndromes of the Aortic Organ. Ann. Thorac. Surg..

[23] Fan Y., Dong J., Wu Y., Shen M., Zhu S., He X., Jiang S., Shao J., Song C. (2022). Development of machine learning models for mortality risk prediction after cardiac surgery. Cardiovasc. Diagn. Ther..

[24] Ong C.S., Reinertsen E., Sun H., Moonsamy P., Mohan N., Funamoto M., Kaneko T., Shekar P.S., Schena S., Lawton J.S. (2023). Prediction of operative mortality for patients undergoing cardiac surgical procedures without established risk scores. J. Thorac. Cardiovasc. Surg..

[25] Allou N., Allyn J., Provenchere S., Delmas B., Braunberger E., Oliver M., De Brux J.L., Ferdynus C., Achouh P., Aubert S. (2023). Clinical utility of a deep-learning mortality prediction model for cardiac surgery decision making. J. Thorac. Cardiovasc. Surg..

[26] Tseng P.-Y., Chen Y.-T., Wang C.-H., Chiu K.-M., Peng Y.-S., Hsu S.-P., Chen K.-L., Yang C.-Y., Lee O.K.-S. (2020). Prediction of the development of acute kidney injury following cardiac surgery by machine learning. Crit. Care.

[27] Behnoush A.H., Khalaji A., Rezaee M., Momtahen S., Mansourian S., Bagheri J., Masoudkabir F., Hosseini K. (2023). Machine learning-based prediction of 1-year mortality in hypertensive patients undergoing coronary revascularization surgery. Clin. Cardiol..

[28] Moor M., Banerjee O., Abad Z.S.H., Krumholz H.M., Leskovec J., Topol E.J., Rajpurkar P. (2023). Foundation models for generalist medical artificial intelligence. Nature.

[29] Acosta J.N., Falcone G.J., Rajpurkar P., Topol E.J. (2022). Multimodal biomedical AI. Nat. Med..

[30] Volovici V., Syn N.L., Ercole A., Zhao J.J., Liu N. (2022). Steps to avoid overuse and misuse of machine learning in clinical research. Nat. Med..

[31] Lundberg S.M., Lee S.I. (2017). A unified approach to interpreting model predictions. Advances in Neural Information Processing Systems 30.

[32] Ribeiro M.T., Singh S., Guestrin C. (2016). ‘Why should I trust you?’: Explaining the predictions of any classifier. Proceedings of the 22nd ACM SIGKDD International Conference on Knowledge Discovery and Data Mining.

[33] Vickers A.J., Elkin E.B. (2006). Decision curve analysis: A novel method for evaluating prediction models. Med. Decis. Mak..

[34] Riley R.D., Ensor J., Snell K.I.E., Harrell F.E., Martin G.P., Reitsma J.B., Moons K.G.M., Collins G., van Smeden M. (2020). Calculating the sample size required for developing a clinical prediction model. BMJ.

